# Elderly offenders at Wathwood Hospital: perspectives and practicalities

**DOI:** 10.1192/bjo.2021.827

**Published:** 2021-06-18

**Authors:** Sidra Chaudhry, Gwilym Hayes

**Affiliations:** Wathwood Hospital

## Abstract

**Aims:**

The following project explores where Wathwood Hospital stands in provision of services to its elderly patients.

**Background:**

The only dedicated forensic medium secure unit for elderly offenders in England is the St. Andrews medium secure unit in Northampton with only 17 beds. Due to the limited beds, other units must accommodate elderly patients, which raises the question whether these units can provide the appropriate services for this very vulnerable population.

**Method:**

Inclusion Criteria:

Male

>55 years of age

Admitted from 2012 onwards (from when database was maintained)

Data were gathered using patient electronic records including index offence, mental disorder, physical health comorbidities and discharge destinations. Patient identifiable data were anonymized to protect their identities.

A staff survey was also conducted to find their perspective on managing elderly patients and whether Wathwood Hospital had the appropriate resources for elderly offenders in their area of work.

**Result:**

A total of 220 referrals were searched with only 9 patients >55 years. Index offenses, mental disorder diagnoses, physical comorbidities including cognitive assessments in the form of memory tests and brain imaging were also collated for identified patients from electronic patient records.

Index offences included violence against person, arson, homicide, robbery, threatening behaviour and dangerous driving and affray. Diagnoses included learning disability, delusional disorder, paranoid schizophrenia, bipolar affective disorder, alcohol dependence, personality disorder and depressive disorder.

Patients had multiple comorbidities such as diabetes, COPD, hypertension, coronary artery disease and musculoskeletal problems. Out of the nine admitted patients, only six had an ACE with an average score of 70.83. Five patients had brain imaging, with two normal results and the others showing some degree of atrophy and ischemic changes.

Discharge destinations included medium secure units, low secure unit and prison. One patient unfortunately died during admission and four are still inpatients.

A staff survey conducted showed their perspective on the challenges in managing elderly patients and whether Wathwood Hospital had the appropriate resources for them to work with elderly offenders in their area of work. All results will be explained through tables and graphs.

**Conclusion:**

It's evident that there are challenges in managing elderly patients in units not specifically designed to manage them. This is also due to the lack of geriatric training and resources available to allied health care professionals to carry out their respective work. It's therefore crucial we formulate more inclusive strategies to address these challenges.

